# Identifying Pathways and Networks Associated With the SARS-CoV-2 Cell Receptor ACE2 Based on Gene Expression Profiles in Normal and SARS-CoV-2-Infected Human Tissues

**DOI:** 10.3389/fmolb.2020.568954

**Published:** 2020-10-16

**Authors:** Qiushi Feng, Lin Li, Xiaosheng Wang

**Affiliations:** ^1^Biomedical Informatics Research Lab, School of Basic Medicine and Clinical Pharmacy, China Pharmaceutical University, Nanjing, China; ^2^Big Data Research Institute, China Pharmaceutical University, Nanjing, China

**Keywords:** SARS-CoV-2, SARS-CoV-2 cell receptor, angiotensin-converting enzyme 2, gene expression profiles, gene set enrichment analysis, gene co-expression network, immune signatures

## Abstract

Because ACE2 is a host cell receptor of the SARS-CoV-2, an investigation of *ACE2* expression in normal and virus-infected human tissues is crucial for understanding the mechanism of SARS-CoV-2 infection. We identified pathways associated with *ACE2* expression and gene co-expression networks of *ACE2* in pan-tissue based on the gene expression profiles in normal human tissues. We found that the pathways significantly associated with *ACE2* upregulation were mainly involved in immune, stromal signature, metabolism, cell growth and proliferation, and cancer and other diseases. The number of genes having a significant positive expression correlation with *ACE2* in females far exceeded that in males. The estrogen receptors (ESR1 and ESR2) and androgen receptor (AR) genes had a significant positive expression correlation with *ACE2*. Meanwhile, the enrichment levels of immune cells were positively associated with the expression levels of *ESR1* and *ESR2*, while they were inversely associated with the expression levels of *AR* in pan-tissue and multiple individual tissues. It suggests that females are likely to have a more robust immune defense system against SARS-CoV-2 than males. *ACE2* was upregulated in SARS-CoV-2-infected tissues relative to normal tissues and in SARS-CoV-2-infected males relative to females, while its expression levels had no significant difference between healthy females and males. Numerous immune-related pathways were highly enriched in SARS-CoV-2-infected males relative to females. These data indicate that males are more susceptible and more likely to have an excessive immune response to SARS-CoV-2 infection than females. This study furnishes potentially cues explaining why females have better clinical outcomes of SARS-CoV-2 infections than males and warrant further investigation for understanding the mechanism of SARS-CoV-2 infection.

## Background

The outbreak of the severe acute respiratory syndrome coronavirus 2 (SARS-CoV-2) has caused a global pandemic (Horton, 2020). This virus has infected more than 34 million people and has caused more than one million deaths as of October 1, 2020 (Covid-19 Dashboard, 2020). The angiotensin-converting enzyme 2 (ACE2) is a host cell receptor of SARS-CoV-2 that plays a crucial role in regulating the SARS-CoV-2 invasion (Hoffmann et al., 2020; Lan et al., 2020; Wang et al., 2020; Yan et al., 2020), which in turn can result in ACE2 upregulation (Smith et al., 2020). Many studies have investigated *ACE2* expression in human tissues (Cao et al., 2020; Li et al., 2020; Liu et al., 2020; Nicin et al., 2020; Zhang et al., 2020a,b). A recent study explored variants in the *ACE2* coding region and the eQTL variants potentially associated with ACE2 expression and compared the related characteristics among different populations (Cao et al., 2020). Our recent study showed that *ACE2* is expressed in a wide variety of human tissues (Li et al., 2020). It suggests that SARS-CoV-2 may attack various human organs, although patients infected with SARS-CoV-2 primarily displayed pneumonia-associated symptoms (Chen et al., 2020; Huang et al., 2020). In this study, we identified pathways associated with *ACE2* expression and gene co-expression networks of *ACE2* in pan-tissue using the datasets from the Genotype-Tissue Expression (GTEx) project (The GTEx Consortium, 2013). We also explored *ACE2* expression in SARS-CoV-2-infected human tissues using a publicly available RNA-Seq dataset. This study aimed to furnish potentially useful cues for further investigation of the SARS-CoV-2 infection mechanism.

## Results

### Pathways and Gene Ontology Associated With *ACE2* Expression

**FIGURE 1:**
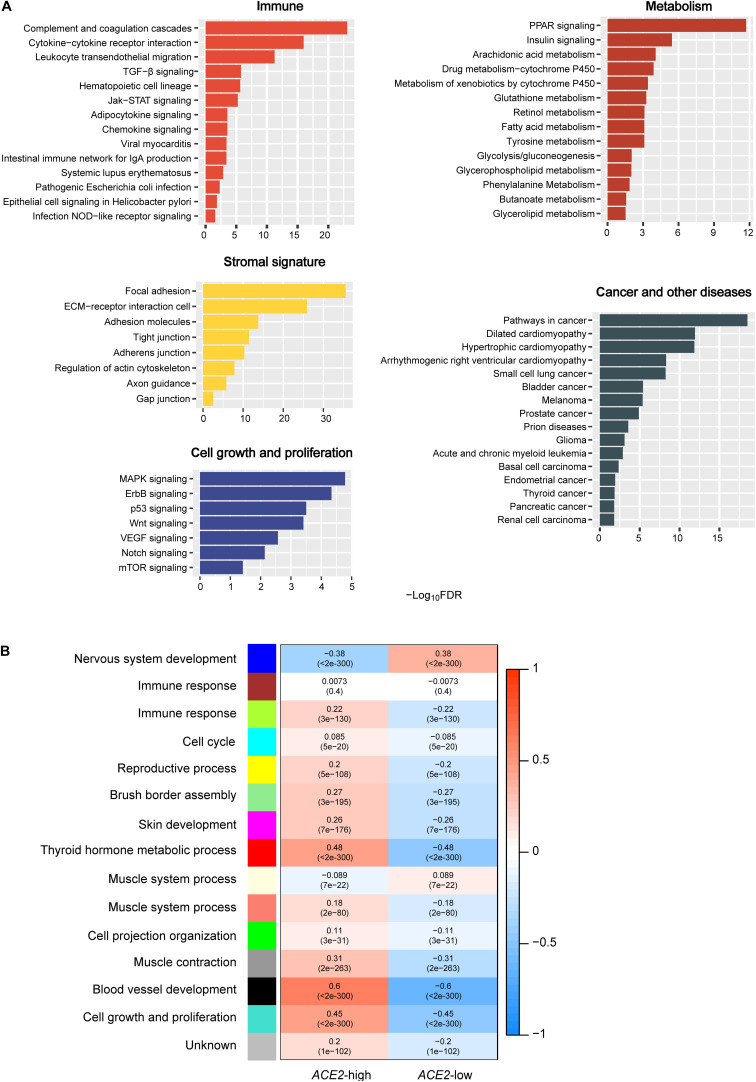
Pathways and gene ontology associated with *ACE2* expression. **(A)** The KEGG pathways significantly associated with *ACE2* upregulation identified by GSEA (Subramanian et al., 2005). **(B)** Gene ontology significantly associated with *ACE2* upregulation and downregulation identified by WGCNA (Langfelder and Horvath, 2008).

We identified highly enriched KEGG pathways in pan-tissue having high *ACE2* expression levels (upper third, sample size *n* = 5,792) versus low *ACE2* expression levels (bottom third, *n* = 5,792) (Student’s *t*-test, adjusted *P*-value FDR < 0.05, fold change (FC) > 2) by GSEA (Subramanian et al., 2005) with a threshold of FDR < 0.05. These pathways were mainly involved in immune, stromal signature, metabolism, cell growth and proliferation, cancer, and other diseases (Figure 1A). The immune-related pathways included cytokine-cytokine receptor interaction, leukocyte transendothelial migration, complement, and coagulation cascades, TGF-β signaling, hematopoietic cell lineage, Jak-STAT signaling, adipocytokine signaling, chemokine signaling, viral myocarditis, intestinal immune network for IgA production, systemic lupus erythematosus, pathogenic Escherichia coli infection, epithelial cell signaling in Helicobacter pylori infection, and NOD-like receptor signaling. The stromal signature-related pathways included focal adhesion, ECM-receptor interaction, cell adhesion molecules, tight junction, adherens junction, regulation of actin cytoskeleton, axon guidance, and gap junction. The metabolism-related pathways included PPAR signaling, insulin signaling, arachidonic acid metabolism, drug metabolism-cytochrome P450, glutathione metabolism, retinol metabolism, fatty acid metabolism, tyrosine metabolism, metabolism of xenobiotics by cytochrome P450, glycolysis/gluconeogenesis, glycerophospholipid metabolism, phenylalanine metabolism, butanoate metabolism, and glycerolipid metabolism. The cell growth and proliferation-related pathways included MAPK, ErbB, p53, Wnt, VEGF, Notch, and mTOR signaling. The cancer-related pathways included pathways in cancer, small cell lung cancer, bladder cancer, melanoma, prostate cancer, glioma, acute and chronic myeloid leukemia, basal cell carcinoma, thyroid cancer, pancreatic cancer, renal cell carcinoma, and endometrial cancer, and the other diseases-related pathways included dilated cardiomyopathy, hypertrophic cardiomyopathy, arrhythmogenic right ventricular cardiomyopathy, and prion diseases.

WGCNA generated 11 gene modules (indicated in green-yellow, cyan, yellow, light green, magenta, red, salmon, green, gray, black, and turquoise color, respectively) that were more highly enriched in the high-*ACE2*-expression-level than in the low-*ACE2*-expression-level pan-tissue (Figure 1B). In contrast, two gene modules (indicated in blue and light-yellow color, respectively) were more highly enriched in the low-*ACE2*-expression-level pan-tissue (Figure 1B). The gene ontology (GO) terms in the highly enriched gene modules in the high-*ACE2*-expression-level pan-tissue were mainly associated with immune response, cell cycle, reproductive process, brush border assembly, skin development, thyroid hormone metabolic process, muscle system process, cell projection organization, muscle contraction, blood vessel development, and cell growth and proliferation. The GO terms in the highly enriched gene modules in the low-*ACE2*-expression-level pan-tissue were mainly associated with nervous system development and muscle system process. The positive associations of *ACE2* expression with immune response, cell cycle, and cell growth and proliferation in pan-tissue were consistent with the pathway analysis results.

### Gene Co-expression Networks of *ACE2*

**FIGURE 2:**
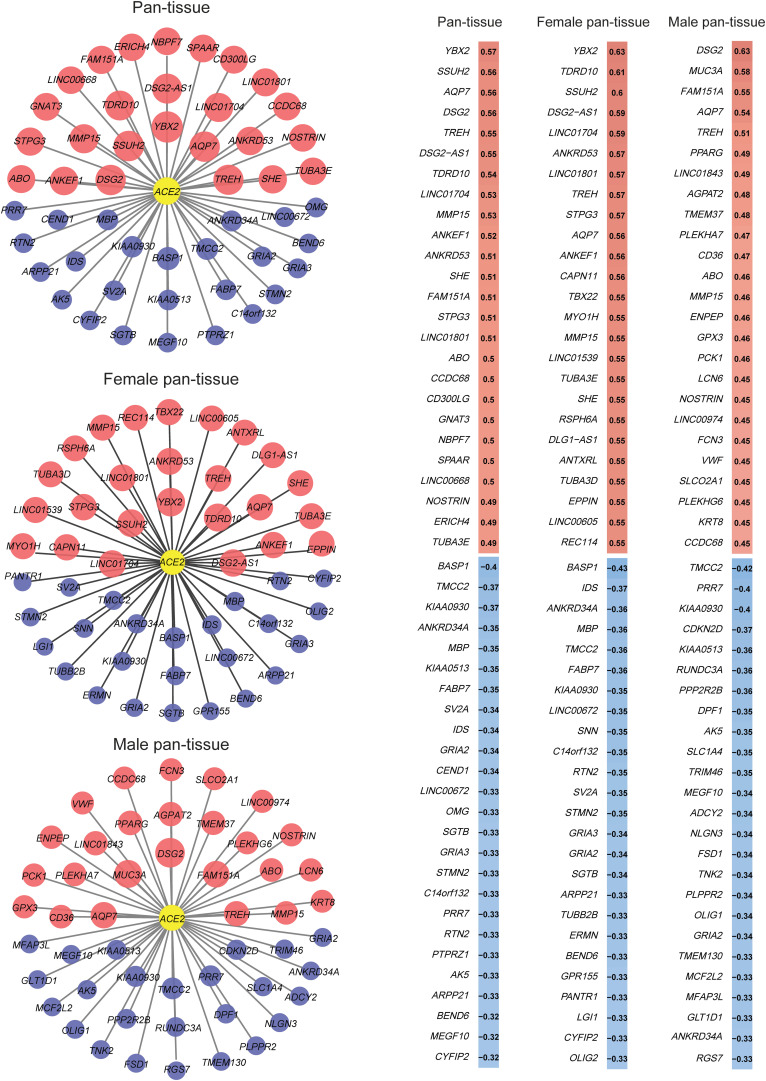
25 genes having the strongest positive and negative expression correlation with *ACE2* in pan-tissue, female pan-tissue, and male pan-tissue. The expression correlation analysis was performed by Pearson’s correlation test, and the gene co-expression networks were generated by Cytoscape (Shannon et al., 2003). The red nodes indicated positive expression associations between the genes and *ACE2*, and the blue nodes indicated negative expression associations. The size of the nodes is proportional to the correlation coefficient.

We found 2,983 and 74 genes having a significant positive and a significant negative expression correlation with *ACE2* in pan-tissue, respectively (Pearson correlation coefficient |*r*| > 0.3) (Supplementary Table 1). Interestingly, when we analyzed female and male pan-tissue individually, we found that 3,940 (or 587) and 87 (or 93) genes having a significant positive and a significant negative expression correlation with *ACE2* in female (or male) pan-tissue, respectively (|*r*| > 0.3) (Supplementary Tables 2 and 3). It indicates that more genes have a significant positive expression correlation with *ACE2* in females than in male pan-tissue. Strikingly, we found 77 genes showing a significant positive expression correlation with *ACE2* in females (*r* > 0.5) but a negative expression correlation with *ACE2* in males (*r* < 0) (Supplementary Table 4). Figure 2 shows 25 genes having the strongest positive and negative expression correlation with *ACE2* in pan-tissue, female pan-tissue, and male pan-tissue (Shannon et al., 2003). Notably, the gene encoding SLC6A19 (solute carrier family 6 member 19), which interacts with ACE2 (Yan et al., 2020), had a significant positive expression correlation with *ACE2* in pan-tissue, female pan-tissue, and male pan-tissue (|*r*| > 0.3).

### Associations of *ACE2* Expression and Immune Signatures With the Expression of Sex Hormone Receptor Genes

**FIGURE 3:**
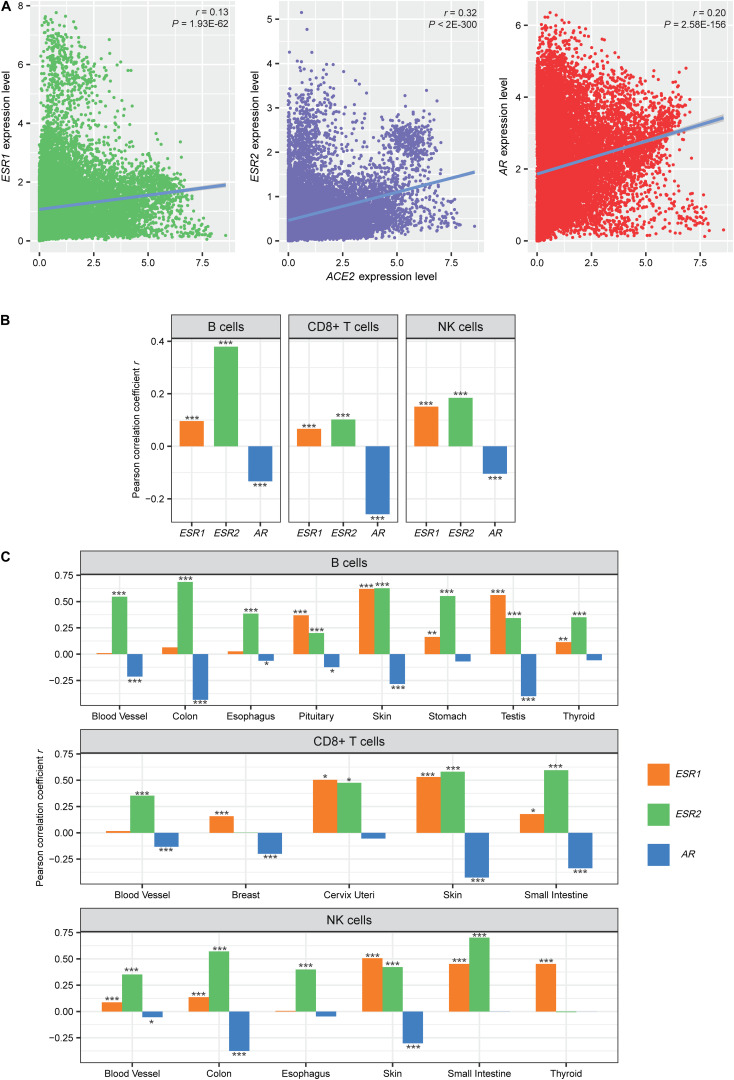
Associations of *ACE2* expression and immune signatures with the expression of sex hormone receptor genes. **(A)** Expression correlations between estrogen receptors (ESR1 and ESR2) and androgen receptor (AR) genes and *ACE2*. **(B,C)** Correlations between the enrichment levels of immune cells and the expression levels of estrogen and androgen receptor genes in pan-tissue and in multiple individual tissues. The correlation coefficient (*r*) and the *P*-value of Pearson’s correlation test are shown. *P < 0.05, **P < 0.01, and ***P < 0.001.

Females have a lower disease severity and mortality risk than males infected with SARS-CoV-2 (Jin et al., 2020; Wenham et al., 2020). A potential explanation is a different host immune response to SARS-CoV-2 infection between females and males (Moulton, 2018; Chen et al., 2020; Gargaglioni and Marques, 2020; Li et al., 2020). We analyzed the correlations between the expression levels of estrogen and androgen receptor genes (*ESR1*, *ESR2*, and *AR*) and *ACE2* expression levels in pan-tissue. We found that the correlations were consistently positive (Pearson’s correlation test, FDR < 1.0 × 10^–60^) (Figure 3A). Interestingly, we found that the expression levels of *ESR1* and *ESR2* were positively associated with the enrichment levels of immune cells (B cells, CD8 + T cells, and NK cells) (Pearson’s correlation test, FDR < 1.0 × 10^–10^) (Figure 3B). In contrast, the expression levels of *AR* inversely correlated with the enrichment levels of these immune cells (FDR < 1.0 × 10^–40^) (Figure 3B). We obtained similar results in many individual tissues, including the blood vessel, breast, cervix uteri, colon, esophagus, pituitary, skin, small intestine, stomach, testis, and thyroid (Figure 3C). These results suggest that females are likely to have a more robust immune defense system against SARS-CoV-2 than males.

### *ACE2* Expression in SARS-CoV-2-Infected Tissues

**FIGURE 4:**
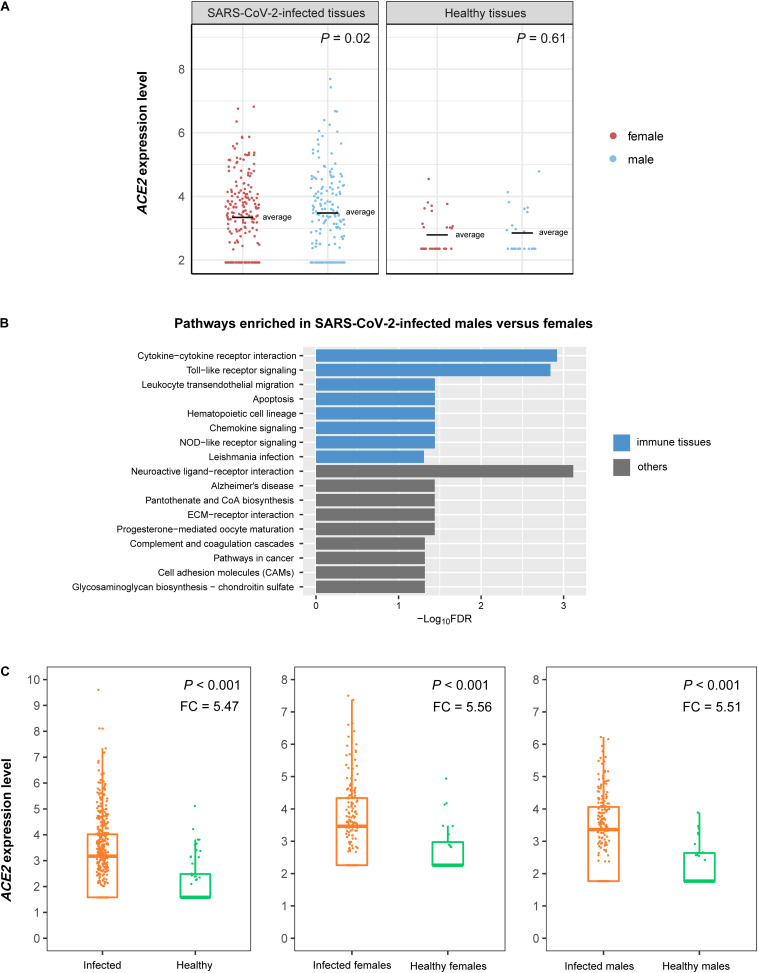
*ACE2* expression in SARS-CoV-2-infected tissues. **(A)** Comparisons of *ACE2* expression levels between SARS-CoV-2-infected males and females and between healthy males and females. **(B)** KEGG pathways highly enriched in SARS-CoV-2-infected males relative to females identified by GSEA (Subramanian et al., 2005). The immune-related pathways are highlighted in blue. **(C)** Comparisons of *ACE2* expression levels between SARS-CoV-2-infected and normal tissues. FC: fold change of mean *ACE2* expression levels.

We found that *ACE2* was more highly expressed in SARS-CoV-2-infected males than in females (*p* = 0.02), while its expression levels showed no significant difference between healthy males and females (*p* = 0.61) (Figure 4A). It suggests that males could be more susceptible to SARS-CoV-2 than females. GSEA identified 17 KEGG pathways highly enriched in SARS-CoV-2-infected males relative to females (Figure 4B). Many were immune-related, including cytokine-cytokine receptor interaction, Toll-like receptor signaling, leukocyte transendothelial migration, apoptosis, hematopoietic cell lineage, chemokine signaling, NOD-like receptor signaling, and Leishmania infection. These results indicate that males are likely to have a higher immune response to SARS-CoV-2 infection compared with females. Furthermore, we found that *ACE2* was more highly expressed in SARS-CoV-2-infected than in normal tissues in both males and females (*p* < 0.001, FC > 5) (Figure 4C). It indicates that SARS-CoV-2 infection may upregulate ACE2 in human host cells that in turn facilitates the SARS-CoV-2 invasion.

## Discussion

**FIGURE 5:**
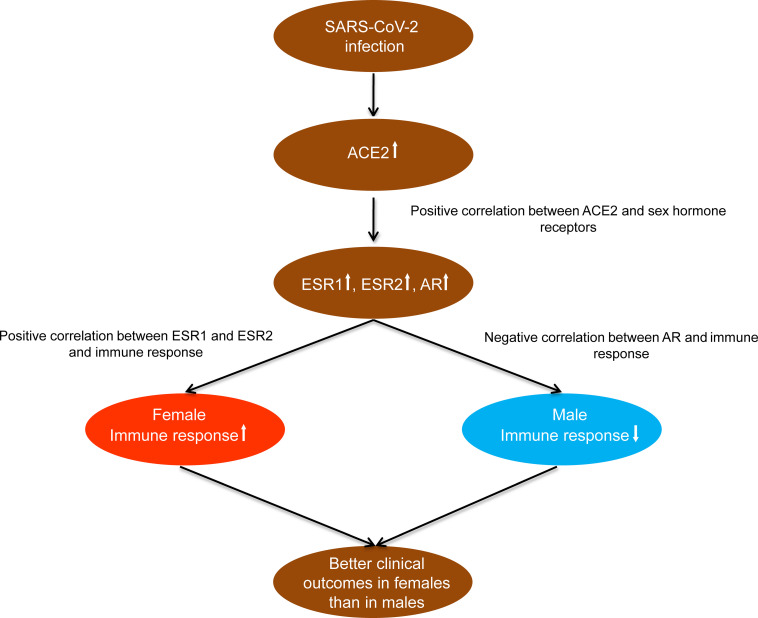
A potential mechanism underlying the significantly different clinical outcomes of SARS-CoV-2 infections between females and males.

By analyzing the gene expression profiles in human tissues, we identified pathways and GO significantly associated with *ACE2* expression. These pathways and GO were mainly involved in immune response, stromal signature, metabolism, cell growth and proliferation, and cancer and other diseases. In particular, the immune response was positively associated with *ACE2* expression, suggesting that the elevated *ACE2* expression may boost the immune response. Indeed, the host adaptive and innate immune responses are crucial in fighting off invading SARS-CoV-2, which uses ACE2 as a host cell receptor (Hoffmann et al., 2020). We found that the enrichment levels of immune cells were positively associated with the expression levels of estrogen receptor genes (*ESR1* and *ESR2*) while they were negatively associated with the expression levels of androgen receptor gene (*AR*) in human tissues. Meanwhile, these sex hormone receptor genes displayed consistent positive expression correlations with *ACE2*. Our recent study showed that *ACE2* expression levels have no significant difference between females and males (Li et al., 2020). Collectively, these results suggest that females have a more robust immune defense system against SARS-CoV-2 than males, partially explaining why females have better clinical outcomes of SARS-CoV-2 infections than males (Figure 5). It also indicates that the supplement with estrogen is a potentially viable approach for treating the patients infected with SARS-CoV-2 (U. S. National Library of Medicine, 2020).

We have also identified gene co-expression networks of *ACE2*. We found that the number of genes having a significant positive expression correlation with *ACE2* in females far exceeded that in males. Again, this difference may provide cues explaining why females and males have markedly distinct severity and mortality risk of SARS-CoV-2 infection. Thus, the genes with a high expression correlation with *ACE2* identified in this study warrant further investigation to understand the SARS-CoV-2 infection mechanism.

Although *ACE2* expression levels had no significant difference between healthy males and females, they were markedly higher in SARS-CoV-2-infected males than in females. Meanwhile, *ACE2* was upregulated upon SARS-CoV-2 infection that in turn may facilitate SARS-CoV-2 infection. The reason why SARS-CoV-2 infection can upregulate *ACE2* to a higher degree in males than in females remains unclear. Moreover, we found that males had a higher immune response to SARS-CoV-2 infection compared with females. This indicates that males are more likely to have an excessive immune response to SARS-CoV-2 infection, the cytokine storm resulting in the immunopathological damage in patients infected with this virus (Li et al., 2020). This could be one of the factors responsible for the worse clinical outcomes in SARS-CoV-2-infected males compared to females.

In analyzing the normal human tissues, we found that females were likely to have a stronger immune defense system against SARS-CoV-2 infection than males. It indicates that females are less susceptible to SARS-CoV-2 infection compared to males. However, in analyzing SARS-CoV-2-infected human tissues, we found that males tended to display a stronger immune response to SARS-CoV-2 infection than females. The reason why SARS-CoV-2-infected males show a stronger immune response than females could be associated with their higher viral loads (Conti and Younes, 2020), as evidenced by the fact that ACE2 is more highly expressed in SARS-CoV-2-infected males than in females. It also could be associated with the compensatory immune-regulatory mechanism (Nish and Medzhitov, 2011). The excessive immune response is likely to lead to a cytokine storm, which may explain why males tend to suffer more severe cases of COVID-19 than females.

## Methods

### Datasets

From the GTEx Project^1^, we downloaded the gene expression profiles (RNA-Seq, TPM normalized) in 30 different human tissues. All gene expression values were added to 1 and then log2-transformed before subsequent analyses. From the NCBI Gene Expression Omnibus database^2^, we downloaded the gene expression profiles in SARS-CoV-2-infected human tissues from nasopharyngeal swabs. A summary of these datasets is presented in Supplementary Table 5.

### Gene-Set Enrichment Analysis

We performed pathway analyses of the differentially expressed genes between the high-*ACE2*-expression-level (upper third) and the low-*ACE2*-expression-level (bottom third) samples in pan-tissue. The differentially expressed genes were identified by Student’s *t*-test using a threshold of FDR < 0.05 and FC > 2. The FDR represented the adjusted *P*-value calculated by the Benjamini and Hochberg method (Benjamini and Hochberg, 1995). Based on the differentially expressed genes, we identified the KEGG (Kanehisa et al., 2017) pathways highly enriched in both groups using GSEA (Subramanian et al., 2005) with a threshold of FDR < 0.05. We used WGCNA (Langfelder and Horvath, 2008) to identify the gene modules (GO) highly enriched in the high-*ACE2*-expression-level and the low-*ACE2*-expression-level samples in pan-tissue. We performed the WGCNA analysis using the R package “WGCNA” (version 1.68).

### Evaluation of the Immune Cell Enrichment Levels in Tissue

We determined the enrichment level of an immune signature in a sample as the mean expression level of the immune signature’s marker genes. Three immune signatures were analyzed, including B cells, CD8 + T cells, and NK cells. The marker genes of these immune cells are presented in Supplementary Table 6.

### Statistical Analysis

We used Pearson’s correlation test to calculate the expression correlations between *ACE2* and other genes in pan-tissue and the correlations between the expression levels of sex hormone receptor genes (*ESR1*, *ESR2*, and *AR*) and the enrichment levels of immune cells (B cells, CD8 + T cells, and NK cells) in pan-tissue and 30 individual tissues. We compared *ACE2* expression levels in SARS-CoV-2-infected and normal tissues between males and females and between SARS-CoV-2-infected and normal tissues using DESeq2 (Anders and Huber, 2010).

## Data Availability Statement

Publicly available datasets were analyzed in this study. This data can be found here: https://www.gtexportal.org/home/datasets/.

## Author Contributions

QF and LL performed data analyses and helped prepare for the manuscript. XW conceived of the research, designed the methods, and wrote the manuscript. All authors read and approved the final manuscript.

## Conflict of Interest

The authors declare that the research was conducted in the absence of any commercial or financial relationships that could be construed as a potential conflict of interest.

## Footnotes

1 https://www.gtexportal.org/home/datasets/

2 https://www.ncbi.nlm.nih.gov/geo/query/acc.cgi?acc=GSE152075

## Supplementary Material

Supplementary Table 1 The list of genes showing a significant expression correlation with *ACE2* in pan-tissue.

Supplementary Table 2 The list of genes showing a significant expression correlation with *ACE2* in female pan-tissue.

Supplementary Table 3 The list of genes showing a significant expression correlation with *ACE2* in male pan-tissue.

Supplementary Table 4 77 genes showing a significant positive expression correlation with *ACE2* in females (Pearson correlation coefficient, *r* > 0.5) but a negative expression correlation with *ACE2* in males (*r* < 0).

Supplementary Table 5 A summary of the datasets used in this study.

Supplementary Table 6 The marker genes of immune cells.

## Abbreviations

ACE2: angiotensin-converting enzyme 2
AR: androgen receptor
ESR1: estrogen receptors 1
ESR2: estrogen receptors 2
GO: gene ontology
GTEx: genotype-tissue expression
NK: natural killer
SARS-CoV-2: severe acute respiratory syndrome coronavirus 2.
